# Study on the spatial decomposition of the infection probability of COVID-19

**DOI:** 10.1038/s41598-023-40307-1

**Published:** 2023-08-15

**Authors:** Lu Liu

**Affiliations:** School of Economics, Southwestern University of Finance and Economics, 555 Liutai Avenue, Wenjiang District, Chengdu, 611130 Sichuan China

**Keywords:** Health care economics, Health policy, Health services, Influenza virus, Viral infection

## Abstract

In the course of our observations of the transmission of COVID-19 around the world, we perceived substantial concern about imported cases versus cases of local transmission. This study, therefore, tries to isolate cases due to local transmission (also called community spread) from those due to externally introduced COVID-19 infection, which can be key to understanding the spread pattern of the pandemic. In particular, we offer a probabilistic perspective to estimate the scale of the outbreak at the epicenter of the COVID-19 epidemic with an environmental focus. First, this study proposes a novel explanation of the probability of COVID-19 cases in the local population of the target city, in which the chain of probability is based on the assumption of independent distribution. Then it conducts a spatial statistical analysis on the spread of COVID-19, using two model specifications to identify the spatial dependence, more commonly known as the spillover effect. The results are found to have strong spatial dependence. Finally, it confirms the significance of residential waste in the transmission of COVID-19, which indicates that the fight against COVID-19 requires us to pay close attention to environmental factors. The method shown in this study is critical and has high practical value, because it can be easily applied elsewhere and to other future pandemics.

Alpha, Beta, Delta, and Omicron, as the many types of variants of the SARS-CoV-2 virus keep emerging, the global pandemic of COVID-19 has developed wave by wave. This is why the information in the first wave of the outbreak is of high importance and value. As this pandemic has evolved into a global disaster, it is considered to be the newest and biggest global health threat^1^, which has already caused serious harm to the sustainability of our cities and society with huge negative impacts.

In the course of our observations of the transmission of COVID-19 around the globe, we have observed considerable concern over distinguishing cases of imported transmission from those of local transmission in a region. If the ratio of imported transmission in the region is high, then the outbreak is relatively easy to contain because we can focus on transportation hubs, such as an international airport. In contrast, if local transmission accounts for a very large proportion, then containment is difficult as it becomes a situation of community spread, which can cause the public to panic. In this study, we try to isolate local transmission from the imported transmission of COVID-19 infection, which can be the key to understanding the pattern in the spread of the disease. Essentially, the most important question here is: to what extent is COVID-19 transmitted locally? To our knowledge, although numerous studies on COVID-19 have emerged since the first wave of the outbreak, this important question has not received enough attention. We, therefore, try to answer this question in this study for the more general purpose of studying any type of infectious disease.

Besides China, the analysis here is also essential for understanding the spread of COVID-19 elsewhere, especially in Europe and the US, as well as in Latin American countries and South Asian countries, where the pandemic has shown a complex pattern in its spread as well. This study also illuminates the way to be against other future pandemics. The remainder of this study is organized as follows. The next section reviews the related literature, and then the data and methods are presented, with a novel spatial analytical framework. The empirical results and a discussion are next, and the conclusion is at the end.

## Related literature

It is really hard to make an overall comprehensive literature review of related studies, as the number of related studies has been huge since the pandemic’s outbreak and many latest studies keep emerging^2–4^. In our study of the spread of COVID-19, we are particularly interested in the environmental factors, which we divide into two. First, we consider a broad concept of environmental factors, which relates to urban studies. They are usually regarded as spatial issues. Second, we consider a narrower concept, that is, hazardous environmental factors such as wastewater and residential waste. Studies on both factors in existing literature cast light on our work.

Concerning spatial issues, some previous studies examine the decline in property values in response to an infectious disease^5^, and spatial resource allocation in an epidemic is also noted^6^. In addition, studies on infectious diseases use a geometric approach^7^, the state-space tracking method^8^, as well as spatiotemporal models^9^. The spatiotemporal models have shown promise in studying COVID-19^10–16^. Unfortunately, not many of them help us understand the probabilities of the COVID-19 outbreak as well as its transmission network among cities. To our knowledge, the spatial pattern of the transmission is somehow overlooked, yet it is a crucial part of fighting against the pandemic^17^.

In fact, there is a long tradition of using spatial models to study environmental issues. Earlier discussions more commonly cover externalities^18^, which is one of the theoretical foundations of environmental economics. Later discussions of carbon dioxide emissions also use spatial analysis^19^. More recent studies mention spatial welfare heterogeneity^20^. In addition, multiregional transboundary pollution is another typical application of the use of spatial effects^21^. Most importantly, studies on biological invasions and their control in the spatial context^22^ show promise in the use of spatial models for the containment of COVID-19. The COVID-19 pandemic is in fact a biological invasion, in the sense that humanity has become a “host” for the SARS-CoV-2 virus, as shown by the confirmation of human-to-human transmission. Therefore, considering the control of a large-scale infectious disease from a wider perspective would be helpful, especially at this unprecedented time. In addition to environmental issues, the use of spatial models is also popular in urban studies^23^ and studies on natural disasters^24^ and crop yields^25^, which offer useful insights into our study.

Moreover, efforts to explore the possible environmental impacts on human health are also long-standing, with earlier discussions most commonly on airborne particulate matter such as PM_10_ and PM_2.5_^26–29^. After the outbreak of COVID-19 worldwide, related discussions in the environmental community gradually grew, and studies on the impact of environmental factors in the transmission of COVID-19 are even described as “an imperative need”^30^. Now, even concerns regarding second or thirdhand smoke have been raised as potential transmission sources^31^. An advanced estimation method for the quantum emission rate has also been introduced^32^.

However, the transmission channel beyond airborne pathways is more easily ignored. Questions have been raised by scholars in water research^33^. In addition, as the SARS-CoV-2 virus has been confirmed to be present in human feces, more environmental implications have been mentioned, such as wastewater systems^34,35^. A pioneer-comprehensive study of the transmission of COVID-19 from an urban perspective identifies both urban wastewater and residential waste as influential factors that can promote the virus’s transmission in cities^36^.

This study proposes a novel explanation of the probability of COVID-19 transmission among the local population in the target city. It hence conducts a spatial statistical analysis of viral transmission, where the transmission of COVID-19 infection among cities is found to have strong spatial dependence. In addition to spatial issues, our analysis examines the role of environmental factors such as wastewater and residential waste in the spread of COVID-19. The significance of residential waste is confirmed in the transmission of COVID-19 in this study.

Unlike other emerging studies, which take a general urban perspective^36^, this study focuses on more technical aspects, primarily in decomposing viral transmission into local and imported components. The spatial models are very important tools to analyze spatial-related issues. Moving from the non-spatial models to spatial models is a big jump in the analytical techniques of COVID-19-related studies, which would help us approach closer to the core part of the issue since the spread of the virus (and hence the pandemic) is essentially spatial. It is not very common, at least not enough, to see the application of this technique in the current pandemic or other large-scale infectious diseases. Besides the application of traditional spatial models, this study also makes some novel contributions in the methodology, which can help analyze the spatial decomposition of the infection probability of COVID-19 as well as other possible large-scale infectious diseases.

## Materials and methods

### Study region and period

Lessons and experiences from China are very valuable for pandemic control in the early stage. In fact, the overlapping periods of the traditional Chinese Spring Festival along with the corresponding national holiday and the epidemic development of COVID-19 in China offer us a “natural experiment” on the evolution pattern of the epidemic. This enables us to isolate the cases of imported transmission from those that were locally transmitted, which is very important in our analysis.

In the first wave of the large-scale outbreak, the COVID-19 epidemic in China can be divided into three stages in early 2020. The first stage is the period (i.e., pre-Jan 24, 2020) before the Spring Festival in China (Chinese Lunar New Year), in which the epidemic gradually worsened in Wuhan before it began to spread just before the national vacation break. The second stage, from January 24 to January 30, 2020, consists of the period of the Spring Festival and vacation, which was later officially extended to February 2, and some companies extended it even further, to February 9. This division occurs naturally, based on the vacation period for the holiday, which involves massive travel comprising the largest scale of human migration in the world. Although the pattern of this temporary migration is rather complicated, in general, it consists of people who work in big cities traveling to their hometowns, which are typically small cities or rural areas, before the holiday, and returning to those big cities after the holiday. From a social perspective, this migration pattern is easily understood, but when the traditional travel pattern is combined with the spread of COVID-19, things can get very complicated.

In the case of China, in the first stage, the COVID-19 infection in China spread from a single epicenter, i.e., Wuhan in Hubei Province, to various locations in China. Wuhan is the epicenter in China, where most cases of COVID-19 infection are confirmed. Other cities in Hubei, of which Wuhan is the provincial capital, can be considered the first ring in the transmission belt in China. Most of the people coming from Wuhan are located in this region. As announced in official reports, more than 5 million people rushed out of Wuhan before the city was locked down^37^. Because of the limited time window and transportation modes, most of them could not go very far, so they remained in other cities in Hubei.

In the second stage, the whole country was alerted about the novel coronavirus spreading, and many cities in China became subject to strict regulations. Therefore, mobility was reduced to a minimal level, as people were asked to stay home to avoid possible infection, which simplifies our analysis. In other words, if new cases of infection with COVID-19 are confirmed at that period, they are most likely due to local transmission from travelers returning from Wuhan before the city was locked down.

However, the real challenge comes in the third stage, when containment of the virus was necessary to avoid further spread. If the people infected with SARS-CoV-2  are in small cities or in rural areas and then return to the big cities across the country, the disease outbreak can evolve from a single epicenter to multiple epicenters, which can lead to a trouble.

**Figure 1:**
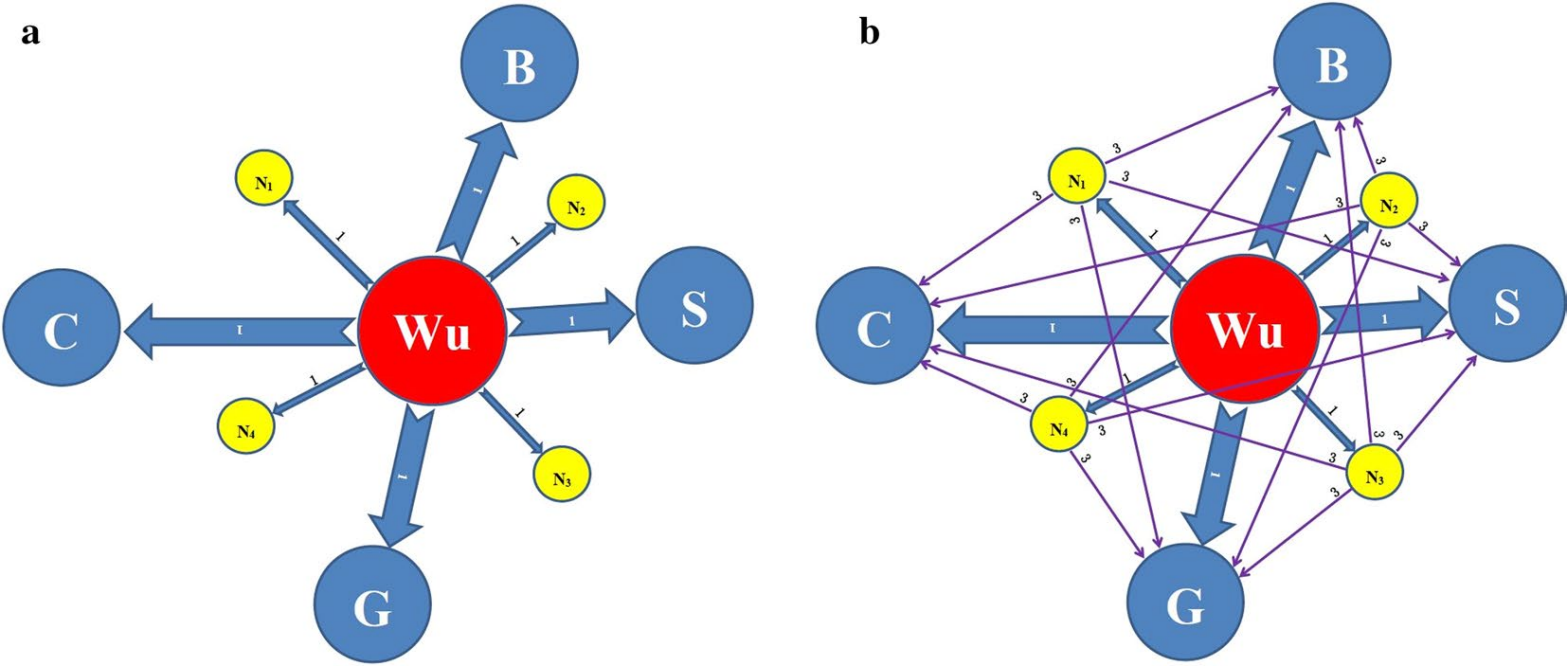
(**a**) Possible transmission patterns of COVID-19 in stages 1 and 2. (**b**) Possible transmission patterns of COVID-19 in stage 3. Possible transmission patterns of COVID-19.

As shown in Fig. 1a, the transmission of COVID-19 in the first stage is relatively simple, with only one epicenter in China, i.e., Wuhan. In this figure, *Wu* is Wuhan, *B* is the metropolitan agglomeration of Beijing and Tianjin, *S* is the metropolitan agglomeration of Shanghai, *G* is the metropolitan agglomeration of Guangzhou and Shenzhen, and *C* is the metropolitan agglomeration of Chengdu and Chongqing. These four urban agglomerations, including the Wuhan region, are the most populated regions in mainland China.

In addition, in Fig. 1 N1–N4 are many midsize and small cities, as well as rural areas. As illustrated in Fig. 1b, the possible transmission pattern of COVID-19 in the third stage becomes much more complicated. With just four examples of small cities shown in the illustration, the transmission network is already very complex. In the real world, small cities might number in the hundreds or even thousands, making the containment of SARS-CoV-2 infection even more difficult. Please note that Fig. 1a,b have no probabilistic meaning or structure, which are not based on real-world data. They are just simple graphical illustrations to show how the pandemic would evolve. Figure 1a,b are just for the general purpose of overall illustration of the transmission pattern, which can be applied to other types of infectious diseases in any other region.

That being the case, why are we so concerned about the second stage? As of January 23, 2020, Wuhan had already been locked down, and if the local transmission in that period is low, then we do not need to worry too much about a possible second wave in China. However, if the transmission in the second stage is severe, then concern over the third stage is warranted.

### Study design

This study matches reported information on the epidemic with the characteristics of cities in China that have COVID-19 cases in the first wave of the outbreak. The introduction of urban characteristics to the analysis of the COVID-19 epidemic has already been discussed comprehensively^36^. In the first wave of the epidemic in China, for most of the cities outside Hubei province, the number of infections became stabilized in early February 2020, so later numbers (many of which are newly imported cases) do not affect our analysis. However, the scale of infection in Hubei province, especially in Wuhan city, is clear until early June 2020 after a city-level PCR test for COVID-19. Therefore, our data set does not include the data in Hubei province to show the spread of infection, which is the right strategy.

To introduce spatial analysis, we add geographic information, such as the GPS coordinates of the cities. We obtain the number of confirmed COVID-19 cases from DingXiang Yuan^38^ at 8:19 a.m. Beijing time, on February 10, 2020. The reason our data are for this particular date is so that we can isolate different scenarios of epidemic transmission in stage 2 from those in stage 3 mentioned earlier. In addition, data on urban characteristics come from the China City Statistical Yearbook 2018^39^. Finally, the GPS coordinates of the cities are from *Google Earth*. The distance between cities is then calculated using the haversine formula, in which the earth’s radius is set as 6371 km^40^.

Table 1 lists the descriptive statistics of all the variables used in this study. We only use data on cities outside Hubei below. Because Wuhan, the capital of Hubei, was the epicenter in China in the first wave of the outbreak, its reported number of COVID-19 cases is lagging due to the technical difficulties in laboratory confirmation in the early stage^36^. This is common in that when a large-scale outbreak occurs, reported cases with symptoms are usually subject to delay^41^.

**Table 1:** Descriptive statistics of variables used (*n* = 296).

Variables	Explanation	Measurement	Mean	Median	Std. dev	Min	Max
*Num_confirmed*	Number of confirmed COVID-19 cases	Cases	34.615	16.000	60.677	1.000	468.000
*Dist*	Distance to Wuhan City	Kilometers	1043.411	905.600	606.808	119.200	3263.100
*Subway*	Length of urban subway	Kilometers	14.540	0.000	69.316	0.000	668.640
*Population_density*	The density of the local population	Persons per square kilometers	3668.179	3035.500	2,406.294	77.000	11,602.000
*Wastewater*	Wastewater discharged annually	10,000 m^2^	13,793.20	5636.500	26,669.229	284.000	229,526.000
*Garbage*	Annual residential waste	10,000 tons	57.540	24.535	103.285	1.560	924.770
*Greenspace*	Public green space per capita	Square meters	14.300	13.680	4.977	2.450	51.660

This table uses data without Hubei Province, where Wuhan is the capital city.

### Statistical analysis

In this section, we apply a set of spatial statistical models. As in statistical techniques dealing with time dependence, spatial statistical models solve correlation problems in space. The two most fundamental and frequently used model specifications are as follows^42,43^.

1 $$y = \rho \times W \times y \, + \, X \times \beta + \, e,$$

2 $$y \, = \, X \times \beta \, + \, u, \, u \, = \lambda \times W \times u \, + \, e.$$

**Figure 2:**
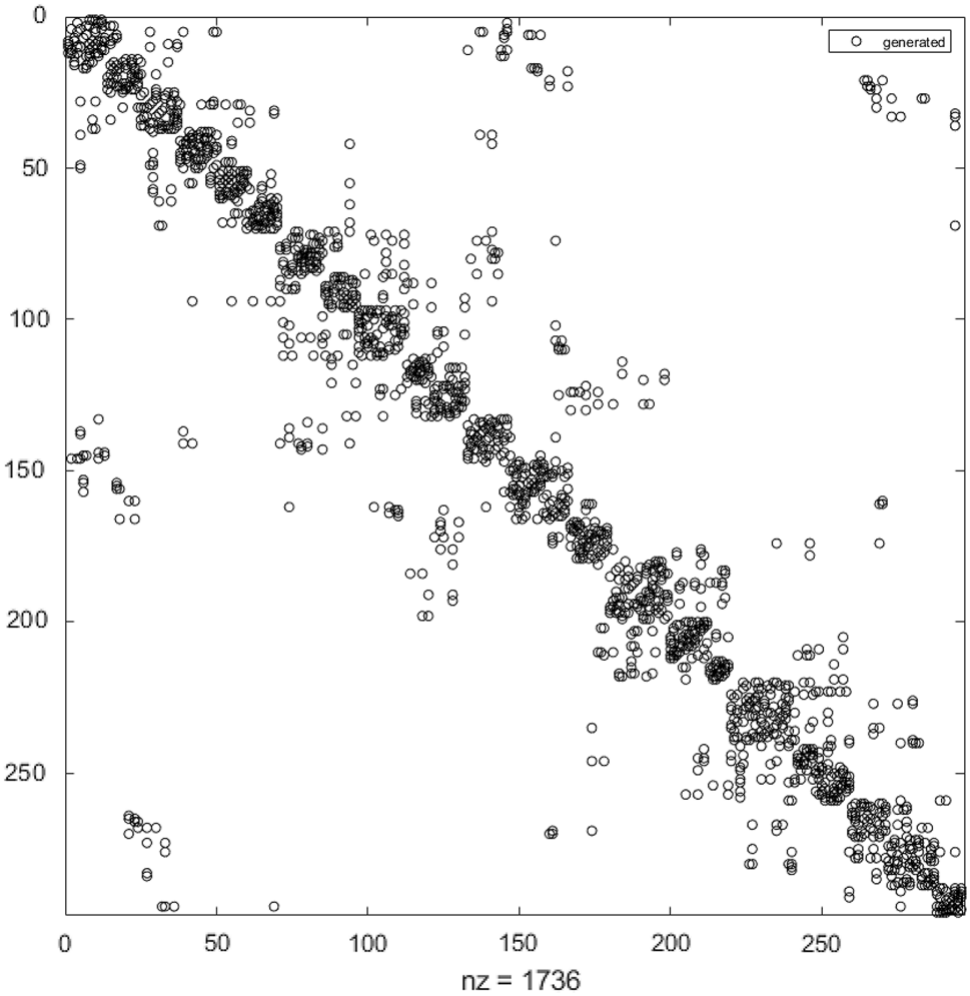
The computer-based simulation of *W.* No cities in Hubei province are included.

Equation (1) is the spatial mixed autoregressive model (SAR), and Eq. (2) is the spatial autoregressive error model (SEM). Although more advanced spatial techniques, such as the three-dimensional spatial weight matrix, were discussed later^40^, these two fundamental model specifications are sufficient for us in this study. These equations are self-explanatory, where *y* is the dependent variable, and *X* is a vector of explanatory variables. *W* is introduced as the spatial weight matrix, which is typically constructed by the reciprocal of the distance between any two cities *i* and *j*, i.e., $1/d_{ij}$, as every element in the matrix. Then, if the data set has *n* cities, the size of *W* must be *n* by *n* (for a computer-based simulation of *W*, see Fig. 2, which is the illustration of the spatial weight matrix that is commonly used in spatial econometrics). Since there can be so many ways of transportation, such as car, bus, train, airline, and even ship, they may result in different time of transportation. Therefore, distance is simple to show the spatial relationship as a general method, which might be more appropriate in our setting than using travel time which is also common in the current GIS technique.

In addition, *e* and *u* are stochastic error terms. Finally, the most important components of these equations are *ρ* and *λ*, which are the spatial dependence of the dependent variable and the error term, respectively. Simply speaking, spatial dependence, also known as the spillover effect, can appear in either the dependent variable or the error term. In practice, the parameters are often calculated with the maximum likelihood estimation (MLE) method or with the generalized method of moments (GMM) as well.

In this study, *y* is the number of confirmed COVID-19 cases. *X* is mainly urban characteristics, including the length of the urban subway, the density of the local population, the wastewater discharged annually, the annual residential waste, and the public green space per capita. Here, the population density is a very close concept to the total population, but it is more meaningful in the analysis of an infectious disease. In addition, the distance to Wuhan might or might not be included in *X*, depending on the performance of the empirical models with different practical meanings. For the convenience of applying probabilities below, here we do not use the log form of the number of COVID-19 cases. However, we do use the log form for many of the explanatory variables for higher precision. Please note that the variables selection here follows Liu^36^, which has successfully built an urban analytical framework for large-scale infectious diseases such as COVID-19. But the analysis in this study is completely new.

As this study tries to isolate local transmission from the imported transmission of COVID-19 infection, we herein propose a novel explanation of the probability of COVID-19 transmission among the local population in the target city based on a spatial statistical analysis of viral transmission.

First, to quantify the spread of COVID-19 among cities in China, we propose the following equation which is self-explained:

3 $$P_{i} = P_{epicenter} \times P_{{{\text{out}}}} \times P_{{{\text{import}}}} \times P_{{{\text{local}}}} ,$$

where *P*_*epicenter*_ is the probability of confirmed COVID-19 cases in the local population at the epicenter of the outbreak, i.e., Wuhan in the case of China in the first wave. *P*_*out*_ is the probability of people’s departure from Wuhan before the city was locked down. *P*_*import*_ is the probability of COVID-19 infection among people in the target city *i* from external sources. In addition, *P*_*local*_ is the probability of the transmission of the novel coronavirus in the target city *i*. Finally, *P*_*i*_ is the probability of confirmation of COVID-19 cases in the local population in the target city *i*.

Equation (3) is the novel contribution of this study. Although it resembles the SEIR models in epidemiology, they are completely different. As we know, in *SEIR* models, *S* means “susceptible,” *E* is “exposed,” *I* stands for “infectious,” and *R* indicates “recovered”. Essentially, it is a combination of four differential equations, which can also be interpreted as the product of multiplying four corresponding probabilities. In this study, as illustrated in Eq. (3), the chain of probabilities is based on the assumption of independent distribution, and this joint distribution is consistent with both logic and common sense. Unfortunately, the probabilities proposed in Eq. (3) are in fact unknown. Therefore, this study tries hard to find some reasonable proxies to simulate these important probabilities. As we can see, *P*_*import*_ and *P*_*epicenter*_ are two steps in the transmission chain. While *P*_*epicenter*_ is completely exogenous in this model, *P*_*import*_ may be affected by several factors that are discussed later in this study. Besides, independent and identically distributed (*i.i.d.*) is a common assumption and practice in setting up the joint distribution, otherwise, the model would become unnecessarily complex.

Second, now the clues are very clear to us. Essentially, Eq. (3) indicates how to calculate the probability of COVID-19 infection in the epicenter of the infection outbreak in a region. Therefore, if we transform Eq. (3), we have:

4 $$P_{{{\text{epicenter}}}} = {\raise0.7ex\hbox{${P_{i} }$} \!\mathord{\left/ {\vphantom {{P_{i} } {P_{{{\text{out}}}} \times P_{{{\text{import}}}} \times P_{{{\text{local}}}} }}}\right.\kern-0pt} \!\lower0.7ex\hbox{${P_{{{\text{out}}}} \times P_{{{\text{import}}}} \times P_{{{\text{local}}}} }$}},$$

which identifies the probabilistic perspective for understanding the scale of the outbreak at the epicenter of the COVID-19 epidemic in that region, which is crucial for us to understand to defeat the virus.

## Results

### Spatial estimation results

We show the empirical estimation results of spatial statistical analysis in Tables 2 and 3. Table 2 includes all the sample cities outside Hubei, showing that the distance between them and Wuhan is a very influential factor in Models (1) to (3). This is evident because Wuhan is the epicenter of the COVID-19 outbreak in China. In addition, in all the models, subways have high statistical significance. We can interpret this from two perspectives. First, subways are a massive method of transportation that can help transmit the virus. Second, cities with a subway system have a high level of urban infrastructure, which makes them attractive to migrants and hence increases the number of infections. Population density, wastewater, and green space are not significant in all the models in Table 2. However, residential waste is found to have a significant impact on COVID-19 infection. The spatial LR and LM tests we conduct here suggest the use of SAR and SEM models, which are shown in Table 2 as well. Please note that the ordinary least square (OLS) estimation results are also presented as a comparison to the spatial models.

**Table 2:** Empirical estimation results with dependent variable *Num_confirmed*, *n* = 296.

	Model (1) OLS (with Dist)	Model (2) SAR (with Dist)	Model (3) SEM (with Dist)	Model (4) OLS (without Dist)	Model (5) SAR (without Dist)	Model (6) SEM (without Dist)
*log(Dist)*	− 21.130*** (− 4.786)	− 16.751*** (− 3.529)	− 20.448*** (− 3.797)			
*Subway*	0.397*** (9.282)	0.401*** (9.541)	0.401*** (9.735)	0.377*** (8.538)	0.390*** (9.163)	0.390*** (9.332)
*log(Population_density)*	1.945 (0.539)	2.710 (0.760)	1.781 (0.496)	3.684 (0.989)	4.323 (1.205)	3.063 (0.837)
*log(Wastewater)*	0.056 (0.010)	− 0.368 (− 0.066)	0.056 (0.010)	3.347 (0.572)	1.599 (0.283)	2.582 (0.451)
*log(Garbage)*	18.390*** (2.737)	18.035*** (2.730)	18.717*** (2.840)	17.401** (2.498)	17.164*** (2.559)	17.347*** (2.583)
*log(Greenspace)*	− 3.913 (− 0.493)	− 4.544 (− 1.663)	− 2.012 (− 0.256)	− 1.928 (− 0.235)	− 3.576 (− 0.452)	− 0.946 (− 0.118)
*ρ*		0.518** (2.240)			0.819*** (7.025)	
*λ*			0.753*** (4.708)			0.879*** (10.628)
Adjusted R^2^	0.506	0.500	0.522	0.469	0.465	0.504
LR tests for spatial correlation in residuals	6.691 (chi-squared value 6.635)			16.043 (chi-squared value 6.635)		
LM error tests for spatial correlation in SAR model residuals		34.260 (chi-squared value 6.635)			81.554 (chi-squared value 6.635)	

The values of the constant terms are not reported. *t* statistics in parentheses. ***p ≤ 0.01, **0.01 < *p* < 0.05, *0.05 < *p* < 0.1.

**Table 3:** Empirical estimation results with dependent variable *Num_confirmed*, *n* = 125.

	Model (7) OLS (with Dist)	Model (8) SAR (with Dist)	Model (9) SEM (with Dist)	Model (10) OLS (without Dist)	Model (11) SAR (without Dist)	Model (12) SEM (without Dist)
*log(Dist)*	− 15.840*** (− 4.327)	− 15.845*** (− 4.392)	− 15.755*** (− 4.014)			
*Subway*	0.374*** (13.058)	0.374*** (13.436)	0.376*** (13.776)	0.386*** (12.657)	0.386*** (13.005)	0.385*** (13.536)
*log(Population_density)*	5.364* (1.658)	5.359* (1.678)	5.253* (1.669)	5.897* (1.702)	6.341* (1.866)	6.069* (1.829)
*log(Wastewater)*	3.734 (0.749)	3.736 (0.771)	2.437 (0.503)	4.116 (0.770)	3.855 (0.742)	2.584 (0.506)
*log(Garbage)*	10.676* (1.917)	10.672** (1.972)	12.268** (2.260)	7.260 (1.229)	7.732 (1.346)	10.132* (1.783)
*log(Greenspace)*	− 6.840 (− 0.935)	− 6.838 (− 0.962)	− 7.161 (− 1.004)	− 2.488 (− 0.320)	− 2.834 (− 0.375)	− 3.637 (− 0.486)
*ρ*		− 0.003 (− 0.009)			0.298 (1.021)	
*λ*			0.515* (1.837)			0.765*** (4.965)
Adjusted R^2^	0.779	0.779	0.783	0.746	0.740	0.762
LR tests for spatial correlation in residuals	1.789 (chi-squared value 6.635)			6.083 (chi-squared value 6.635)		
LM error tests for spatial correlation in SAR model residuals		3.490 (chi-squared value 6.635)			26.441 (chi-squared value 6.635)	

The values of the constant terms are not reported. *t* statistics in parentheses. ***p ≤ 0.01, **0.01 < *p* < 0.05, *0.05 < *p* < 0.1.

In addition, in models (4) to (6), we omit the variable for distance to Wuhan to examine whether the spatial weight matrix *W* sufficiently reflects spatial dependence. In these models, the spatial tests produce much higher values than the models that include the variable for the distance to Wuhan. As we can see, distance matters a lot in spatial statistical models.

To improve the predictive power of the models, we delete many “remote” cities with few reported COVID-19 cases. This refinement yields a subsample with fewer but more representative observations. The adjusted *R*^2^ values are much higher in Table 3, which suggests a better model fit, and the population density variable becomes significant. However, the *ρ* values are less significant in the SAR models. Finally, the *λ* values are still significant in the SEM models, which means that the error terms still have spatial dependence issues even after the data refinement.

Since the available information is very limited, this study has to consider a restricted set of covariates. However, as shown in Table 3, the adjusted *R*^2^ can be up to 0.783, which is a very good overall performance.

### Calculation of the infection probability at the epicenter

Now the most essential question in this study arises: among the five probabilities listed in Eq. (3), which are already known? Which are obtainable by estimation? And which are still unknown?

The number of confirmed COVID-19 cases in any target city outside Hubei, especially Wuhan, is the known information. Hence *P*_*i*_ can be considered as given, so it might appear as if the final answer is already presented. However, the epidemic conditions of COVID-19 are not so simple. As noted earlier, we do not know the exact number of COVID-19 cases in Wuhan in the early or even middle stage (of the first wave) of the epidemic, thus *P*_*epicenter*_ is considered unknown.

The probability of people’s departure from Wuhan before the city was locked down, i.e., *P*_*out*_ is much easier to determine because it was officially reported that 5 million out of 14 million people left Wuhan before it was locked down^5^. Therefore, we can set *P*_*out*_ = 5/14.

When people left Wuhan, they could have gone to several possible locations, which is relevant to *P*_*import*_. But measuring this probability is technically difficult. The simple way to do it is to use the reciprocal of the distance between Wuhan and the destination city because the shorter the distance between that destination city and Wuhan, the more easily people could travel between them, which results in a larger value of *P*_*import*_. However, this approach relies on the assumption of a uniform distribution. In reality, that destination city might be far from Wuhan, as the reasons that some people went to particular places are unknown. Thus, using the spatial statistical techniques shown above is a more precise way to measure this probability. As noted, *ρ × W × y* shows the spillover effect of spatial dependence, which identifies the spread of the virus from all other cities except itself. Therefore, we can denote *P*_*import*_ as follows:

5 $$P_{{{\text{import}}}} = {\raise0.7ex\hbox{${\hat{\rho }Wy}$} \!\mathord{\left/ {\vphantom {{\hat{\rho }Wy} {Dist}}}\right.\kern-0pt} \!\lower0.7ex\hbox{${Dist}$}},$$

where the hat (caret) above the variable means the estimated value and *Dist* is the distance from the target city to Wuhan. As shown, the numerator of Eq. (5) measures the spillover effect of the amount of COVID-19 infection from outside the target city, and the denominator indicates the physical difficulty of traveling among cities.

Finally, the probability of the local transmission of COVID-19 is similarly unclear. Even the latest technology in virology and epidemiology has not yet clarified exactly how the virus is transmitted within a city. Case studies (i.e., epidemiological investigations) have even more difficulty in doing so because of the infinite possibilities in person-to-person and surface-to-person transmission. However, the spatial techniques presented above can isolate locally explained factors that influence the infection with COVID-19. Because *X* represents independent variables for the local characteristics of the target city, $X\hat{\beta }$ can be considered the locally explained factors that contribute to COVID-19 infection in the target city. Thus, here we construct the following equation:

6 $$P_{local} = {\raise0.7ex\hbox{${X\hat{\beta }}$} \!\mathord{\left/ {\vphantom {{X\hat{\beta }} y}}\right.\kern-0pt} \!\lower0.7ex\hbox{$y$}},$$

where the numerator is the model-based amount of infection that is locally explained, and the denominator is the actual number of confirmed COVID-19 cases in the target city.

Please note that Eqs. (5) and (6), though they are both ratios, have completely different meanings. While Eq. (5) shows how the epidemic transits to another city, Eq. (6) presents only how it spreads locally in the target city. Here we need to clarify that “locally explained factors” include people who left the epicenter and were later confirmed as COVID-19 cases since they may spread the virus to others in the destination city. In this way, spatial statistical methods enable us to achieve a goal that is difficult and costly to attain using case studies. These discussions and calculations provide the technical ability to estimate the probability of COVID-19 infection in the epicenter of the outbreak. As illustrated in Eq. (4), we can calculate the probability of infection in Wuhan from the perspective of every sample city in the data set, denoted by *P*_*epicenter_i*_, where the subscript *i* is the city in the sample. Because every city in the data set has an “opinion” on how many people are infected in the epicenter, we need to calculate the mean value of these “ideas” as follows.

7 $$P_{{{\text{epicenter}}}} = \sum\limits_{i = 1}^{N} {P_{{{\text{epicenter\_i}}}} }.$$

According to Eq. (4), we can calculate a corresponding value for each city. Therefore, we need to calculate an average value for all the cities, which becomes Eq. (7).

Using all the information above, we obtain a value of *P*_*i*_ as 0.00315%, *P*_*out*_ is given as 5/14, *P*_*import*_ is calculated as 1.651%, and *P*_*local*_ is 188.812%, and all are averages. Because of the setting in Eq. (6), *P*_*local*_ can be more than 1, just as a ratio. So, the final calculated result is *P*_*epicenter*_ = 0.009243. Probabilities are essentially ratios, at least a major one of the many concepts. As we can see, some of the middle-step ratios may have a value that is greater than 1, but the final result is consistent with the definition of probability.

## Discussion

### Estimation of the extent of infection in the epicenter

The remainder of this discussion is straightforward. If we know the probability of infection in Wuhan, we can arrive at a very good estimation of the exact number of infections in the city by multiplying this probability by the city’s population. The last question that remains is the size of the population to use in the final calculation.

If we use the total population of Wuhan, the predicted number of infections is likely to be too high because the population distribution in a city is not uniform. Typically, closer to the center of the city, the population is denser. Thus, we divide the population of Wuhan into three core districts, known as the three towns of Wuhan: Hankou, Wuchang, and Hanyang. These three towns account for only about 4.331% of the total area of Wuhan including rural areas, but their aggregate population is 4.477 million, which is about 31.978% of the total population of Wuhan^44^. In particular, this area includes Hankou, the most populated district in Wuhan. Therefore, we tend to use the population of these three core towns to represent the total population of Wuhan. As a result, the estimated infection number of COVID-19 is 41,378.129. As of June 16, 2020, the actual number of confirmed COVID-19 cases in Wuhan in the first wave is reported to be 50,340. Therefore, our estimation in this study is reasonable.

### More possible contributions in spatial models

A notable feature of the empirical results shown earlier is that our results suggest the non-constant *ρ* and *λ*, which is against the traditional spatial models as Eqs. (1) and (2) present. This finding itself may shock the fundamental assumption of spatial econometrics since we are dealing with the variant version of *ρ* and *λ*. In addition, in both Tables 2 and 3, we see that models without “*Dist*” have higher values of *ρ* and *λ*, which can be explained by the negative sign of the partial derivatives of $\frac{\partial \rho }{\partial Dist}$ and $\frac{\partial \lambda }{\partial Dist}$. As we see, the expression “without ‘*Dist*’” can be equivalent to “*Dist* = 0”, which implies higher values of *ρ* and *λ* if the previous partial derivatives are negative. Therefore, the derivatives here can be the breakthrough in the methodology, i.e., the theory of spatial econometrics. However, we are not able to do so at this moment given the limited information we have. Although this study does not give rigorous mathematical proof of such negative signs in the derivatives, future studies can follow this hint.

### Rethinking the critical role of lockdown

Although we have derived the probability of infection as well as the corresponding size at the epicenter of an outbreak (in this case, of Wuhan), we can still draw more policy implications from our discussion. Recall Eq. (3), which shows the multiplication of four probabilities, and also illustrates the transmission chain of COVID-19. If we want to contain the epidemic by reducing *P*_*i*_, we should cut off the chain of transmission by reducing the corresponding probability on the right-hand side of Eq. (3). However, changing *P*_*epicenter*_ is not feasible because it is determined by unknown factors that are exogenous to our model. Therefore, the feasible policies rely on the other three probabilities: *P*_*out*_, *P*_*import*_, and *P*_*local*_.

First, the lockdown of Wuhan and later all of Hubei proved essential in containing the outbreak at the first stage of transmission, as mentioned earlier; otherwise, *P*_*out*_ would be much larger. The lockdown helped to reduce *P*_*import*_ as well. Moreover, social distancing, as well as regulations at the community level in nearly the entire country, helped to reduce *P*_*local*_ to a large extent. In China, these policies together successfully prevented the COVID-19 epidemic from developing into the third stage in the first wave of the outbreak, as mentioned earlier, which would have had catastrophic results. In fact, after the outbreak of COVID-19 in China in the first wave, the most critical window of opportunity for China to effectively contain the epidemic in the early stage was only seven days, based on the following. This point is confirmed by a government official in a speech about the two years of the lockdown of Wuhan, which is considered to be the “decisive move” in China’s fight against COVID-19 in the first wave of the outbreak^45^. The lockdown of Wuhan on January 23, 2020, was followed by a weeklong national holiday for Spring Festival. If people across the whole country had returned to work after this date, without this weeklong pause, perhaps nothing could have prevented the infection from developing into the third stage, as shown in Fig. 1b, making it much more difficult to contain it. Unfortunately, this is what happened elsewhere.

Moreover, our results discussed in “More possible contributions in spatial models” also demonstrate the hints that the closer the target city is to the epicenter, the larger the “spillover effect” would be for virus transmission among neighboring cities. Such a clue may suggest the policy implication that to contain the rapid spread of the virus, we do not only need to focus on the epicenter itself but also need to pay close attention to the surrounding cities nearby.

### Policy implications

Now, the numbers of COVID-19 are still chalking up records every day around the world. Under this background, this study has proposed a method that tries to isolate cases due to local transmission from those due to externally introduced COVID-19 infection through spatial regression models. This study proposes an important method, although it is not perfect, it is still useful. It makes the following contributions. First, we decomposed the transmission of COVID-19 into spillover and local effects. Second, we use a probabilistic perspective to analyze the scale of the first wave of the outbreak at the epicenter of COVID-19 in a region.

Although many aspects of COVID-19 continue to be revealed, one thing that is already evident is that cutting off the chain of transmission at the earliest opportunity is of the highest importance. Therefore, as the pandemic continues to develop rapidly in many places, this study can provide timely analysis to help understand the local transmission pattern of COVID-19 and hence save lives.

In a study, He et al. conclude from a cross-sectional sample that “6.92% of the population of Wuhan developed antibodies against SARS-CoV-2”^46^. It in fact shows the infection probability in Wuhan, China as we have mentioned in this study. However, such antibody tests and examinations are very costly and time-consuming, and they cannot provide a prompt evaluation of the infection probability in the early stage of an outbreak. In addition, the number of test samples is still limited, and the result may be biased due to the biased distribution of test samples in the population. Therefore, the method proposed in this study provides valuable and fast estimation in the early stage of the outbreak of any wave, which can be much faster than the study of antibodies against SARS-CoV-2. And the result of our calculation is reasonable, which is verified by the real-world data.

If a researcher or policymaker wants to examine the spatial characteristics of a pandemic such as COVID-19 or any other infectious disease, the spatial statistical model can be a useful tool to examine how the virus transmits in space (i.e., the spatial dimension or context). The method we use in the paper to calculate the probability of infection in the first wave of the outbreak in Wuhan, China, can easily be applied elsewhere for any wave with a slight adjustment in the setup of the probabilities. For example, by constructing a similar spatial structure for analysis, we can conduct a similar study on Europe and the US for any target city. Thus, the method shown in this study is of broad interest and high practical value. Since the ongoing pandemic comes wave by wave, and each time the model presented in this study can be helpful. This study is not perfect, but it may show some directions for future studies. This study is merely a simple try, yet it has already demonstrated the capacity and potential of the spatial techniques to contribute to the analytical framework of the spread of the pandemic. This model is also useful in the sense that it can be applied to the city-level analysis, in which both the infection zone and the possible quarantine zone can be studied. In this scenario, some part (or district) of a city can be considered to be the epicenter of a wave of outbreak, and other parts of the city can be considered to be the target zones. Therefore, the method shown in this study is still applicable.

In addition, in some other studies^35,36^, although both wastewater and residential waste are found to have a strong impact on the transmission of COVID-19, only residential waste is confirmed with its significance in this study. This is the usual outcome when the model settings are substantially different. Although this study introduces spatial attributes, its result also suggests that residential waste may be statistically more significant than wastewater, as it is significant even after a big change in the model setup. In real cases, perhaps the only confirmed source of infection related to residential waste is a small wave of local COVID-19 epidemic in November 2020 in Chengdu, China, which is due to a leak in the garbage disposal in a quarantine site as the official investigation reported^47^. Also, in February 2022, three environmental sanitation workers are infected in Huhhot, China, the clue of which again leads to residential garbage. But this clue is not confirmed yet^48^. However, the true mechanism between the waste and the spread of COVID-19 at the city level is yet unknown, which can be considered one of the limitations of this study. Though is it no longer significant, the sign of wastewater in this study is still generally positive, which is consistent with our expectations as well. Therefore, during the fight against COVID-19, we need to pay closer attention to urban wastewater and residential waste, where one or more of the key factors of the transmission of COVID-19 may be hidden^49^.

## Conclusion

It has been more than three years, and the COVID-19 pandemic is still ongoing. However, humanity needs to look forward, and we eventually need to find a way to get along with it. Now, the sustainable development^50^ of humanity has already been greatly challenged by the COVID-19 pandemic. As the virus prevails everywhere at the neighborhood level^51^, more environmental parameters^52^ need to be considered and hence strict waste management^53^ must be implemented.

This study can be considered as a retrospection. Thus, this study has direct application value, and it is also of general interest to readers of public health or related fields. Our results are also important for public officials as they try to respond to the rapidly spreading pandemic accurately and immediately. Indeed, although the virus and the corresponding pandemic are scientific issues, eventually they become an issue of management and public administration.

On May 5, 2023, World Health Organization (WHO) announces that COVID-19 is no longer a public health emergency of international concern (PHEIC)^54,55^. As the COVID-19 pandemic is gradually becoming history (at least we hope so), some scholars may think that the significance of the finding of this study is less since it focuses on the COVID-19 pandemic management which is not a concern now. However, the virus is still out there^35^, and its corresponding epidemic may come in a seasonal manner^49^ or mini-waves^56^.

In addition, we also would like to see how this study can help against future pandemics with a special focus on the use of quantitative methods. Hopefully, it may inspire many other cutting-edge insights and knowledge to a wide range of topics that relate to the social and economic impacts of the COVID-19 pandemic both in and post-pandemic era. It may also shed further light on the way to construct social resilience against the COVID-19 pandemic or other future pandemics.

### Limitations and future research topics

This study still has several limitations. First, although we constructed a spatial weight matrix among cities, a deeper level of spatial relationship among neighborhoods within cities would be more informative. Second, the precision of the models could be improved by using simulation-based methods and a larger sample size. Third, given the limited available data, we could not include more environmental factors in this study. Fourth, as mentioned earlier, this study does not address the issue of the variant version of *ρ* and *λ* as well as the negative sign of their derivative with respect to the distance to the epicenter with formal proof in math. Fifth, time matters. Therefore, temporal factors need to be included to set up the complete spatiotemporal analytical framework. These limitations could be addressed in future research. Finally, the analytical framework shown in this study can also be useful in more simulations to justify the efforts in this work as well as those after relaxing the zero-policy facing Omicron^57^. All these can be done in future studies.

## Data Availability

No human subjects are involved. All other data used in this study are publicly available. Please see the references for details.
